# Improved Chinese Giant Salamander Parental Care Behavior Detection Based on YOLOv8

**DOI:** 10.3390/ani14142089

**Published:** 2024-07-17

**Authors:** Zhihao Li, Shouliang Luo, Jing Xiang, Yuanqiong Chen, Qinghua Luo

**Affiliations:** 1 School of Computer Science and Engineering, Jishou University, Zhangjiajie 427000, China; zhli2501@163.com; 2 Hunan Engineering Laboratory for Chinese Giant Salamander’s Resource Protection and Comprehensive Utilization, School of Biological Resources and Environmental Sciences, Jishou University, Zhangjiajie 427000, China; luosl2021@163.com (S.L.); xiangjing@ccsu.edu.cn (J.X.); 3 Hunan Engineering Technology Research Center for Amphibian and Reptile Resource Protection and Product Processing, College of Biological and Chemical Engineering, Changsha University, Changsha 410022, China; 4 College of Biology and Pharmacy, Yulin Normal University, Yulin 537000, China; 5 School of Computer Science and Engineering, Central South University, Changsha 410017, China

**Keywords:** *Andrias davidianus*, parental care behavior, YOLOv8s, multi-scale convolution

## Abstract

**Simple Summary:**

This study initially analyzed surveillance videos to identify and extract key moments of *Andrias davidianus*’ parental care behavior and established a dataset for this behavior. Based on this, this study optimized the existing YOLOv8 object detection model specifically for this task and proposed the ML-YOLOv8 model, which shows outstanding performance in recognizing and analyzing *A. davidianus*’ parental care behavior. Following testing and verification, the ML-YOLOv8 model demonstrated excellent performance in efficiently and accurately detecting *A. davidianus*’ parental care behavior. The findings of this study not only provide evidence for optimizing breeding technology and conservation management of *A. davidianus* in their natural habitat but also offer new technical means and research ideas for studying amphibian behavioral ecology.

**Abstract:**

Optimizing the breeding techniques and increasing the hatching rate of Andrias davidianus offspring necessitates a thorough understanding of its parental care behaviors. However, *A. davidianus*’ nocturnal and cave-dwelling tendencies pose significant challenges for direct observation. To address this problem, this study constructed a dataset for the parental care behavior of *A. davidianus*, applied the target detection method to this behavior for the first time, and proposed a detection model for *A. davidianus*’ parental care behavior based on the YOLOv8s algorithm. Firstly, a multi-scale feature fusion convolution (MSConv) is proposed and combined with a C2f module, which significantly enhances the feature extraction capability of the model. Secondly, the large separable kernel attention is introduced into the spatial pyramid pooling fast (SPPF) layer to effectively reduce the interference factors in the complex environment. Thirdly, to address the problem of low quality of captured images, Wise-IoU (WIoU) is used to replace CIoU in the original YOLOv8 to optimize the loss function and improve the model’s robustness. The experimental results show that the model achieves 85.7% in the mAP50-95, surpassing the YOLOv8s model by 2.1%. Compared with other mainstream models, the overall performance of our model is much better and can effectively detect the parental care behavior of *A. davidianus*. Our research method not only offers a reference for the behavior recognition of *A. davidianus* and other amphibians but also provides a new strategy for the smart breeding of *A. davidianus*.

## 1. Introduction

The Chinese giant salamander (*Andrias davidianus*) is one of the largest and oldest existing amphibians in the world. It is a flagship species of endangered amphibians and a rare species unique to China [1]. In China, only the wild *A. davidianus* belongs to grade II protected animal, and the artificially cultured *A. davidianus* can be reasonably utilized [2]. The nutritional value of *A. davidianus* is high, which is rich in 8 essential amino acids required by the human body. It has good development and utilization potential in the fields of food, health care, medicine, etc. and is known as “Water Ginseng” [3,4]. In 1978, the first artificial breeding of *A. davidianus* was successful in Sangzhi County, Zhangjiajie, Hunan Province [5]. After years of research and successful breeding practices, artificial breeding of *A. davidianus* emerged and developed rapidly in the late 1990s. However, although the artificial breeding and breeding technology of *A. davidianus* has increasingly matured, it still faces many technical bottlenecks, especially the low level of breeding technology and high cost of fry, which greatly limit the economic benefits of the *A. davidianus* industry. Among them, the parental care behavior of *A. davidianus* is crucial for the species’ survival and procreation. The occurrence of this behavior can increase the dissolved oxygen content in the egg hatching water environment, provide the necessary oxygen for embryonic development, and directly affect the hatching rate of the egg. In addition, it can effectively reduce the risk of water mold infection in offspring and further improve the hatching success rate [6,7]. Therefore, the in-depth study on the juvenile protection behavior of *A. davidianus* not only helps us to understand its biological characteristics but also provides valuable theoretical support for solving the problems in the breeding technology of *A. davidianus*.

Currently, the monitoring of the parental care behavior of *A. davidianus* predominantly depends on manual observation. The breeding behaviors of *A. davidianus* are acquired through infrared surveillance systems, and, subsequently, a detailed analysis of the recorded videos was conducted [6,7]. This methodology is not only time consuming and demands specialized knowledge but is also prone to subjective biases in handling the extensive data processing involved, posing considerable challenges to animal behavior research.

In the domain of object detection, there are two classic types of algorithms: two-stage object detection algorithms and one-stage object detection algorithms. The two-stage object detection algorithms include the region-based convolutional neural network (R-CNN) [8], fast R-CNN [9], and faster R-CNN [10]. The one-stage object detection algorithms encompass the YOLO series of algorithms (you only look once) [11,12,13,14,15,16,17], and the SSD algorithm (single shot multibox detector) [18]. Two-stage object detection methods require generating numerous candidate boxes and classifying the objects within them, whereas single-stage object detection methods can directly obtain the positions of the target boxes, thereby avoiding the generation of candidate boxes. In practical applications, the one-stage object detection method not only accelerates the detection process but also enhances the overall performance in real-world scenarios.

With the advancement of deep learning, many researchers have begun to apply target detection methods to analyze behaviors such as nursing, rolling, and gathering in animals [19,20,21]. Xu et al. [22] used the model faster R-CNN to realize the behavior identification of fish under different ammonia concentrations. Xue et al. [23] optimized faster RCNN by designing a new residual structure for the backbone network and introducing supervisory signal center loss to construct a joint classification loss function, which improved the accuracy and speed of faster RCNN’s behavior detection of nursing sows. Based on the idea of the SKNet [24] network, Wen et al. [25] proposed an adaptive scale attention mechanism to optimize the faster R-CNN model, which enhanced the feature extraction capability of multi-scale complex targets. Kang et al. [26] used the lightweight network MobileNet v2 [27] to replace the original feature extraction network VGG-16 and enhanced the receptive field through the variable convolutional layer to improve the detection effect of the SSD model on abnormal behavior of the crowd. Hu et al. [28] optimized the YOLOv3 model by improving the pooling block and loss function, which improved the accuracy and performance of YOLOv3 on fish behavior. Wang et al. [29] added a small target detection frame to carry out multi-scale fusion to optimize the YOLOv5 model and improve the detection accuracy of abnormal behavior of porphyry bream. Tu et al. [30] added a large-size detection head in YOLOv8 and then invoked the ECAM attention mechanism to reduce the interference of fuzzy background, focus on the key features of individual fish, and enhance the recognition ability of fuzzy fish.

Despite the application of target detection methods based on deep learning in the field of animal behavior, recognition is quite widespread and has achieved remarkable results. However, there is a significant gap in the application of this technology to the recognition of amphibian behaviors, with research on the behavior recognition of *A*. *davidianus* remaining largely unexplored. Therefore, this study proposes a parental care behavior detection method for *A. davidianus* based on the YOLOv8s algorithm. The primary contributions of this study are as follows:

1. For the first time, a deep learning model was used to automatically identify *A. davidianus*’ parental care behavior, optimizing the method of observing *A. davidianus*’ behavior, promoting the application of information technology in amphibian behavioral ecology research, and providing a reference for the study of other amphibians or aquatic animals;
2. We constructed the first dataset dedicated to the behavior of amphibians, *A. davidianus*’ parental care behavior dataset. This dataset includes six fundamental behaviors: tail fanning, agitating, shaking, egg eating, entering caves, and exiting caves;
3. Inspired by the concepts of Res2Net [31], this study proposes a multi-scale feature fusion convolution (MSConv), which is integrated with the C2f module to form C2f-MSConv. Experimental results demonstrate that this module significantly enhances the model’s feature extraction capability and reduces computational costs;
4. The integration of the large separable kernel attention (LSKA) [32] mechanism in the SPPF layer minimizes background interference in *A. davidianus*’ parental care behavior detection. Additionally, the WIoU [33] loss function addresses issues of error and missed detections associated with low-quality samples.

## 2. Materials and Methods

### 2.1. Materials

#### 2.1.1. Data Collection

**Figure 1:**
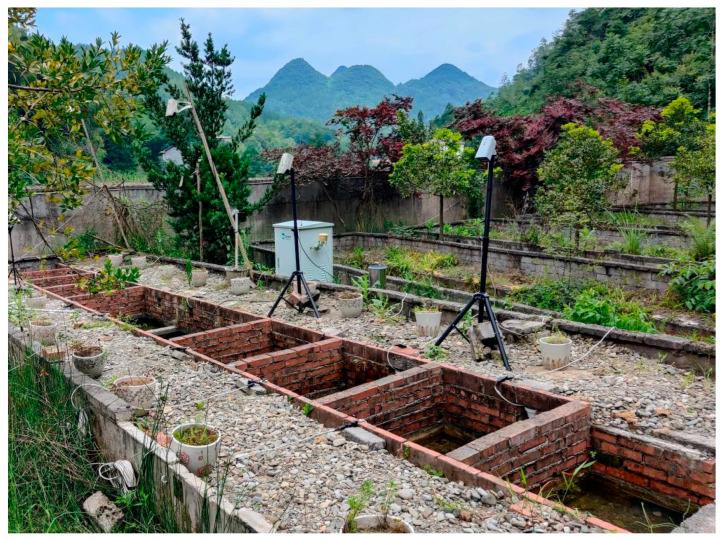
Simulate natural *A. davidianus*’ breeding pond.

The surveillance video of four pairs of *A. davidianus* was collected in the simulated natural breeding pool of Zhangjiajie Zhuyuan Giant Salamander Biotechnology Co., Ltd. (29°25′56″ N, 110°22′55″ E, altitude: 471 m) in Tangxiyu Village, Kongkeshu Township, Sangzhi County, Zhangjiajie City, Hunan Province, during the period from August to October annually between 2020 and 2022. The simulated natural breeding pool in the base consists of artificial streams and caves, which are distributed on both sides of the artificial streams (Figure 1).

#### 2.1.2. Dataset Creation

**Figure 2:**
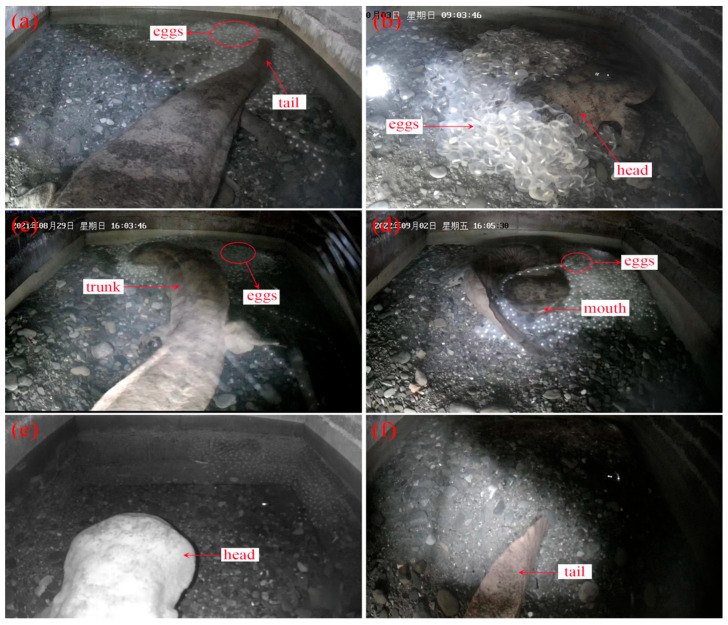
Example of *A. davidianus*’ parental care behavior dataset: (**a**) tail fanning; (**b**) agitating; (**c**) shaking; (**d**) egg eating; (**e**) entering caves; (**f**) exiting caves.

Using video editing software (Format Factory 5.13), the keyframes of parental care behavior of *A. davidianus* in the breeding period were randomly screened and intercepted from the video to make an image dataset. The quality of images in the dataset will significantly affect the training effectiveness of the model, and it is necessary to check the numerous captured images and remove inferior ones. Finally, the behavior dataset of this experiment was obtained, including six behaviors, namely, tail fanning (600), agitating (700), shaking (500), eating eggs (700), entering caves (250), and exiting caves (250), and the dataset images were manually defined and labeled by the “LabelImg” image labeling tool. The evaluation criteria for each behavior are shown in Table 1, and examples of behaviors are shown in Figure 2. The data were randomly selected and divided into training sets and test sets in a ratio of 8:2.

### 2.2. Standard YOLOv8 Model

**Figure 3:**
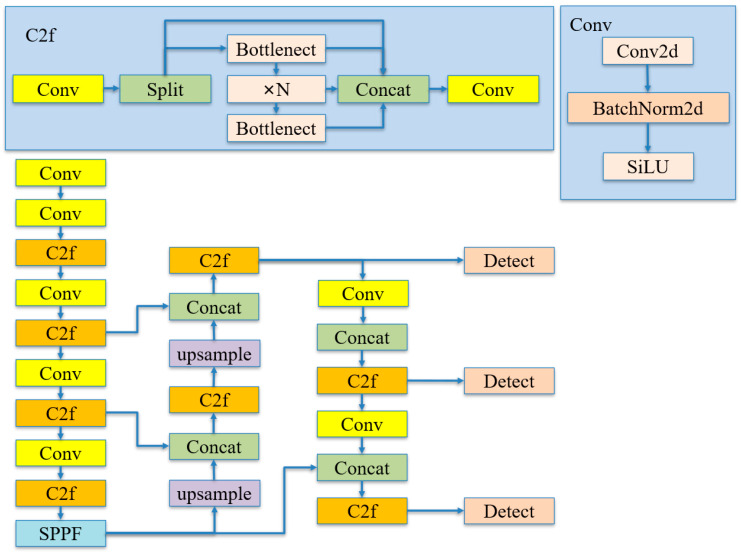
Standard YOLOv8 model structure diagram.

The YOLOv8 algorithm is a fast single-stage object detection method composed of input segment, backbone segment, neck segment, and output segment. From large to small, there are five versions: nano, small, middle, large, and extra-large. With the increase in model size, model accuracy continues to improve. Based on the hardware constraints of edge devices, the YOLOv8s model with small size and high precision is selected in this study. The standard YOLOv8 network is shown in Figure 3, and the technical terms are shown in Table A1.

The input segment is used for data enhancement and adaptive image scaling. The primary method of data enhancement is Mosaic augmentation, which is being introduced in the Yolov8 model by adopting the Mosaic augmentation technique that is used in the final 10 epochs of YoloX. This technique enhances the robustness of the model. Additionally, adaptive image scaling is utilized to uniformly resize the original images to a consistent dimension, simplifying the model’s complexity and effectively improving its accuracy.

The backbone segment is composed of modules such as Conv, C2f, and SPPF. The Conv module, which primarily includes Conv2d, batch normalization (BN), and Swish activation function, is responsible for performing convolution operations on feature maps. The C2f module serves as the main component for feature extraction. The SPPF (spatial pyramid pooling fast) module is used for extracting features from different receptive fields. This architectural design enables the network to better adapt to targets of varying scales.

The neck segment incorporates the FPN–PAN structure to enhance the model’s feature fusion capability. FPN [34] (feature pyramid network) is top-down. Through up-sampling, the feature information of the upper layer is fused with the feature information of the lower layer to calculate the predicted feature map. PAN [35] (path aggregation network) is an improvement of FPN, introducing horizontal connections to enhance the semantic information of features so that bottom-up feature maps can be fused with top-down feature maps.

The head segment employs an anchor-free matching mechanism, which requires only the regression of the center points and the width and height of the targets in feature maps of different scales. This approach significantly reduces the time consumption. Ultimately, by leveraging rich information from feature maps of various scales, it accurately obtains the classification and location information of target objects of large, medium, and small sizes.

### 2.3. Improved YOLOv8 Model

**Figure 4:**
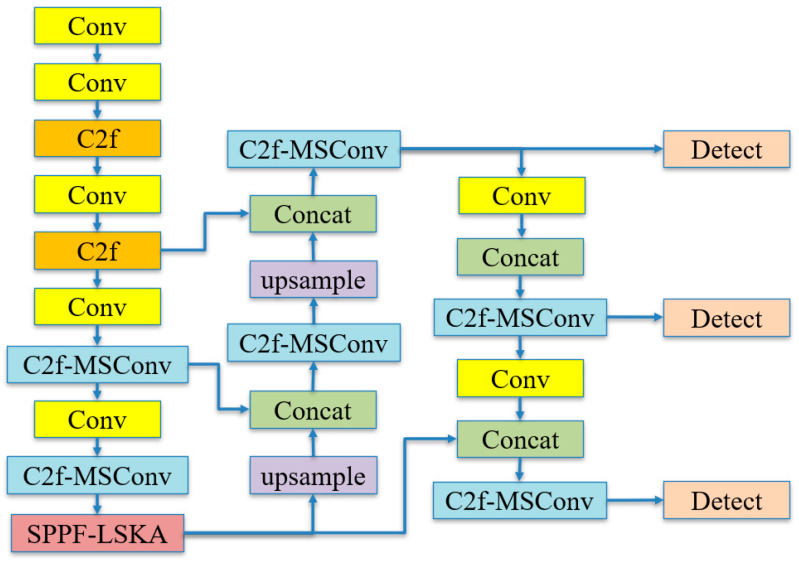
Improved YOLOv8 model structure diagram.

The improved structure of YOLOv8s, as shown in Figure 4, has been enhanced as follows: Firstly, we propose a multi-scale convolutional (MSConv) combined with C2f module to obtain C2f-MSConv, which replaces some C2f modules in the network structure and enhances feature extraction capability. Secondly, we introduce the LSKA attention mechanism in the SPPF module to reduce the interference of irrelevant background features on target detection, thus improving the accuracy of the model. Finally, we replace the original CIoU bounding box loss function with Wise-IoU.

#### 2.3.1. Multi-Scale Convolution C2f-MSConv Module

Multi-scale features have always been an important aspect of detection tasks. Since the introduction of zero convolution, multi-scale pyramid models based on zero convolution have achieved milestone results in detection tasks. The information about an object captured under different receptive fields is different. A small receptive field can capture more details of the object, which is also very beneficial for detecting small targets. In contrast, a large receptive field can capture the overall structure of the object, facilitating the network’s localization of the object’s position. Combining details and position can better extract clear object information.

**Figure 5:**
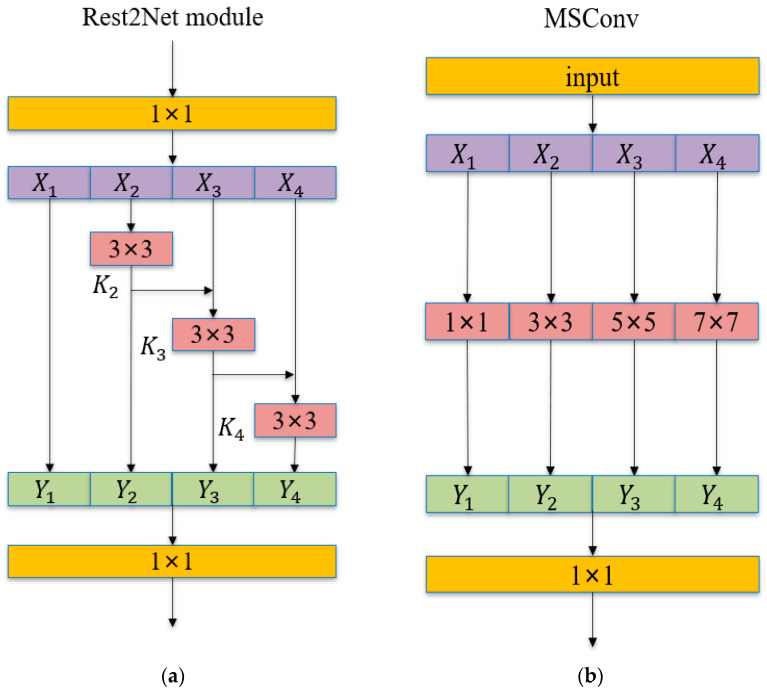
(**a**) Res2Net module; (**b**) MSConv structure.

The core structure of Res2Net is shown in Figure 5, the feature $K_{2}$ is processed through a 3 × 3 convolutional layer, which is positioned at $X_{3}$ within the module’s architecture. Following this initial convolution, $K_{2}$ undergoes a second optimization through another 3 × 3 convolution, effectively simulating the impact of a 5 × 5 convolution through the combination of two successive 3 × 3 convolutions. Subsequently, $K_{3}$ is enhanced by integrating the processed features from both the 3 × 3 and the compounded 5 × 5 receptive fields. Similarly, the feature $K_{4}$ is subjected to a 7 × 7 receptive field, further expanding the scale of feature detection. The formula is shown below:

(1) $$Y_{i}=\left\{\begin{array}{ll} X_{i} & i=1;\\ K_{i} & i=2;\\ K_{i}(X_{i}+Y_{i-1}) & 2<i\leq s. \end{array}\right.$$

Based on the concept of Res2Net, we designed a new type of MSConv (multi-scale convolution). As shown in Figure 5, we use grouped convolutions to divide the original input channel count into four parts and extract features at different scales through 1 × 1, 3 × 3, 5 × 5, and 7 × 7 convolutional operations. Finally, a 1 × 1 convolutional layer is used to fuse multi-scale features, achieving comprehensive feature extraction and efficient integration.

By combining MSConv with the C2f module to propose C2f-MSConv, which replaces some of the original C2f in YOLOv8, not only is the feature redundancy significantly reduced and the efficiency of feature extraction improved but also the features of different scales are fully utilized, thereby further enhancing the performance of the neural network.

#### 2.3.2. Optimization of Feature Fusion Networks

**Figure 6:**
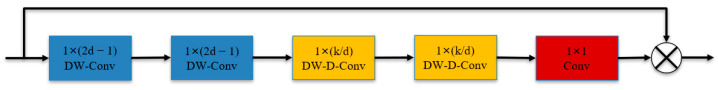
LSKA attention structure.

**Figure 7:**
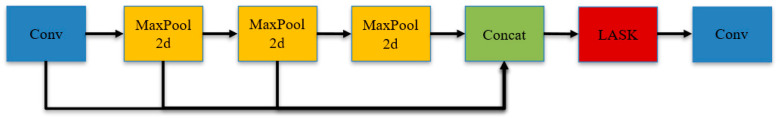
SPPF-LSKA module structure.

When constructing a deep learning model for recognizing the behavior of the *A. davidianus*, considering the complexity and variability of the environment in which the *A. davidianus* lives, to enhance the model’s ability to recognize and extract key features of the *A. davidianus*’ behavior, we have introduced a type of attention mechanism called LSKA (Figure 6) into the spatial pyramid pooling fast (SPPF) of YOLOv8 (Figure 7). This mechanism is designed to help the network filter out irrelevant background information, thereby focusing more on capturing effective feature information related to the behavior of the *A. davidianus*.

**Figure 8:**
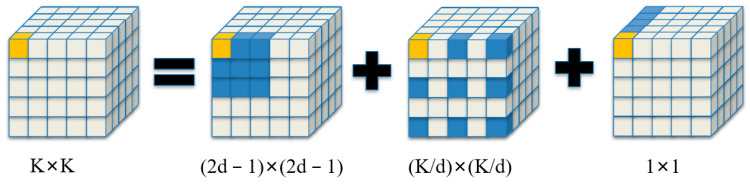
Large kernel convolution decomposition process.

**Figure 9:**
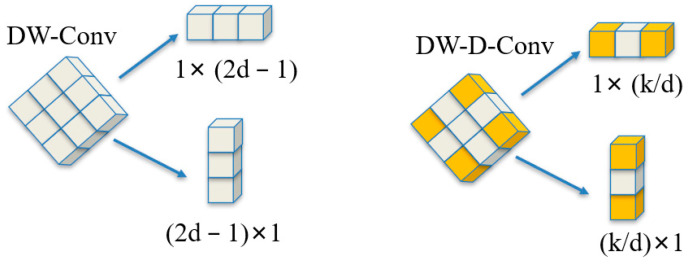
2D Convolution decomposition into 1D process.

Compared with traditional attention mechanisms such as self-attention and large kernel attention (LKA) [36], LSKA has been innovatively designed. Although the self-attention mechanism excels in handling long-range dependencies and adaptability, it often overlooks the two-dimensional structural characteristics of images. To address this issue, the large separable attention (LSA) mechanism was proposed, which improves the accuracy of feature extraction by considering the two-dimensional structure of images. However, LSA faces the challenge of excessive computational load when dealing with large-sized convolutional kernels. The LSKA mechanism effectively overcomes the limitations of LSA by decomposing large-sized convolutional kernels, achieving high performance at a lower computational cost. Specifically, LSKA first decomposes a K×K convolutional kernel into (2d−1)×(2d−1) depthwise convolution, (K/d)×(K/d) depthwise dilated convolution, and 1×1 convolution (Figure 8). Then, it further decomposes these 2D convolutional kernels and depthwise dilated convolution kernels into 1D horizontal convolutional kernels and vertical convolutional kernels (Figure 9) and then connects these decomposed convolutional kernels in sequence to form an efficient attention module.

This innovative structure not only optimizes the computational efficiency of the model but also improves the accuracy of recognizing the behavior features of the *A. davidianus*, providing more precise technical support for the analysis of the behavior of the *A. davidianus*.

#### 2.3.3. Improved Regression Loss Function

In the field of object detection, the YOLOv8 model employs CIoU (complete intersection over union) as its default loss calculation method. The CIoU loss function not only considers the overlapping area between the predicted bounding box and the ground truth bounding box but also introduces the distance and aspect ratio between the two, allowing the loss function to pay more attention to the shape characteristics of the bounding box. However, when collecting and annotating data on edge devices, due to environmental and conditional limitations, the obtained data often include some low-quality samples. The presence of these low-quality samples, if punished using traditional geometric measurements, will overly amplify their impact, leading to a decline in the model’s generalization performance.

To address this issue, the improved network model adopts a new bounding box regression loss function—WloU [33]. The core idea of the WloU loss function is to dynamically reduce the punishment for geometric measurements when the overlap between the anchor box and the target box is high. This approach enables the model to maintain good generalization capabilities even when facing low-quality data collected on edge devices. The expression of the WloU loss function is as follows:

(2) $$L_{IoU}=1-\frac{B_{box}\cap T_{box}}{B_{box}\cup T_{box}};$$

(3) $$R_{WIoU}=exp\left(\frac{{\left(x_{b}-x_{t}\right)}^{2}+{\left(y_{b}-y_{t}\right)}^{2}}{{\left(W^{2}+H^{2}\right)}^{*}}\right);$$

(4) $$L_{WIoU}=R_{WIoU}L_{IoU}.$$

**Figure 10:**
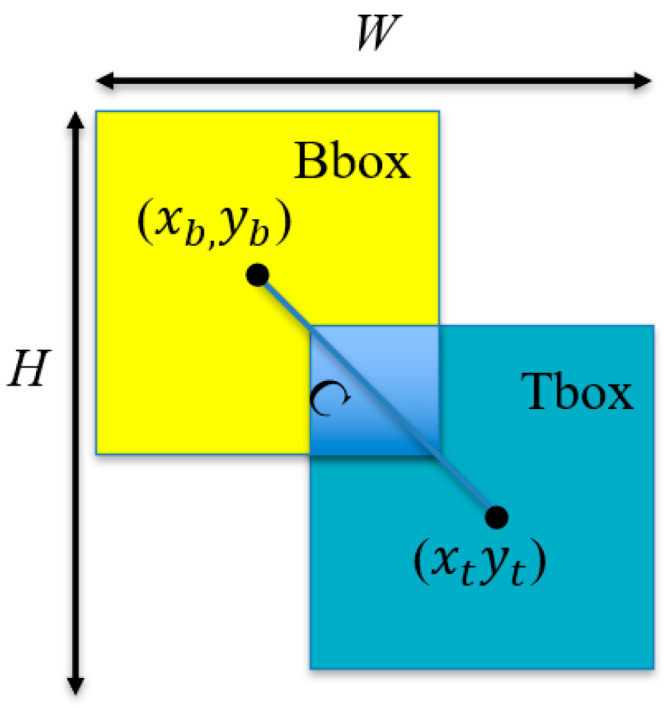
Definition of relevant parameters.

The values of *W*, *H*, ( $x_{b}$ , $y_{b}$ ), and ( $x_{t}$ , $y_{t}$ ) are illustrated in Figure 10.

In the above formula, *W* and *H* represent the width and height of the bounding box formed by the predicted box and the true box. The asterisk (*) indicates that the width and height of the smallest enclosing box are excluded from the gradient calculation to reduce the adverse impact on model training.

## 3. Results

### 3.1. Experimental Environment and Parameter Adjustment

The experimental operating system utilized in this study was Ubuntu 20.04, with Pytorch serving as the framework for developing deep learning models. Detailed specifications of the experimental environment are outlined in Table 2. Input images were standardized to a size of 640 × 640, and the batch size was set to 16, with training being conducted across 300 epochs. The learning rate used during model training was 0.01, with an SGD momentum of 0.937 and an optimizer weight decay of 0.0005. All other training parameters were set to the default values of the YOLOv8 network.

### 3.2. Assessment of Indicators

To provide an objective assessment of the performance of *A. davidianus* behavior detection models, the evaluation metrics employed encompass GFLOPS (giga floating-point operations per second), which quantifies the execution time of the network model in terms of billions of floating-point operations per second. The parameters, which assess the size and complexity of the model. FPS (frames per second), which gauges the detection speed of the model in frames transmitted per second. mAP (mean average precision), utilized to evaluate the model’s accuracy.

TP represents the true positives (the number of target frames that are correctly predicted to be in the positive category), FP represents the false positives (the number of target frames that are incorrectly predicted to be in the positive category), and FN represents the false negatives (the number of target frames that are actually in the positive category but are incorrectly predicted to be in the negative category).

Precision is the ratio of the number of target boxes correctly predicted by the model as positive categories to the number of all target boxes predicted by the model as positive categories and is defined as

(5) $$Precision=\frac{TP}{TP+FP}.$$

Recall is the ratio of the number of target frames correctly predicted as positive categories by the model to the number of target frames in all actual positive categories and is defined as

(6) $$Recall=\frac{TP}{TP+FN}.$$

AP is the area under the precision–recall curve and represents the average precision of the model at different recall rates. It is defined as

(7) $$AP=\int_{0}^{1}Precision\;d(Recall).$$

mAP is a comprehensive metric used to assess the performance of object detection models across multiple categories. It calculates the average precision (AP) for each category and then takes the average of these AP values to gauge the model’s performance.

(8) $$mAP=\frac{\Sigma_{i=1}^{N}AP\left(i\right)}{N}$$

N represents the number of categories in the dataset, and N in this study is equal to 6. The higher the mAP value, the better the model’s performance. Precision and recall are dimensionless measures expressed as a ratio of the number of correct predictions to the total number of predictions, typically represented as a percentage.

### 3.3. Comparison of Ablation Experiments

For this section, based on the *A. davidianus* dataset, ablation experiments were conducted to explore the improvement effects on the overall model. Starting with YOLOv8s as the base model, we sequentially modified it by replacing C2f with C2f-MSConv, introducing the LSKAattention mechanism into the SPPF layer, and adopting the WIoU loss function. The experimental results of the models on *A. davidianus* dataset are shown in Table 3.

Referring to Table 3, the following can be seen:

1. Through the comparative analysis between the first and second sets of experiments, we found that the proposed MSConv module demonstrated significant advantages on this dataset. It not only effectively reduced the model’s parameter count and computational load (measured in GFLOPs) but also successfully improved the model’s mAP;
2. Further, in the comparison between the second and third sets of experiments, we introduced the LSKA mechanism into the SPPF layer. Although this led to a slight increase in the model’s parameter count and computational load, it significantly enhanced the model’s ability to extract feature behaviors in complex backgrounds, resulting in a 0.5% increase in mAP;
3. Lastly, by comparing the third and fourth sets of experiments, we replaced the original model’s CIoU loss function with the WIoU loss function. The dynamic gradient distribution strategy of WloU inhibits the learning of low-quality samples and improves the mAP by 0.3%.

**Figure 11:**
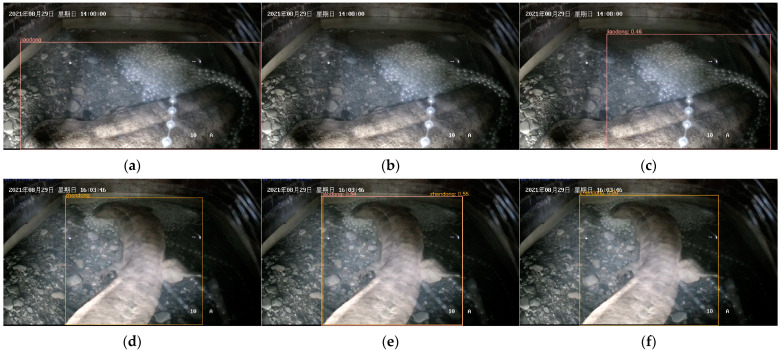

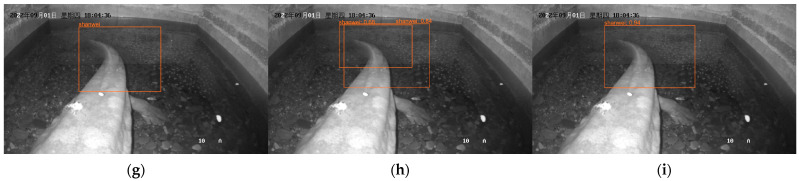
Figures (**a**,**d**,**g**) are the annotation boxes of the dataset. Figures (**b**,**e**,**h**) are the detection results of original model. Figures (**c**,**f**,**i**) are the detection results of the improved model for the same image.

**Figure 12:**
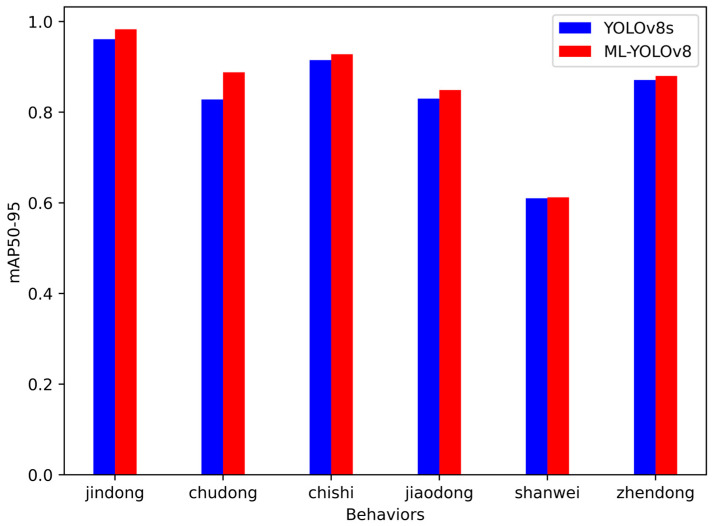
mAP comparison of six behaviors before and after improvement.

To provide a clearer visual comparison of the detection capabilities between the original and the improved models, Figure 11 and Figure 12 illustrate the *A. davidianus* detection outcomes. The left is the annotation in the dataset, the middle is the detection result of YOLOv8s, and the right is the detection result of ML-YOLOv8.

Based on the comparative analysis through Figure 11 and Figure 12, it is evident that YOLOv8 exhibits instances of missed detections and false positives during the detection process. In contrast, the improved model proposed in this study is capable of accurately detecting the behavior of *A. davidianus* complex backgrounds. Furthermore, our method significantly enhances the confidence level of the detected *A. davidianus*’ behaviors. An evident improvement can be observed in the average accuracy assessment for each behavior.

### 3.4. Comparison Experiments

To assess the performance of the enhanced model, this study conducted comparative experiments between the enhanced model and various widelyused object detection models. The chosen models encompass two-stage anchor-based approaches, including faster R-CNN, one-stage anchor-based approaches like SSD, YOLOv5, and YOLOv7 and one-stage anchor-free models YOLOX. The experiments were carried out on the same dataset and under identical experimental conditions.

It can be observed from Table 4 that the algorithm proposed in this study exhibits remarkable mAP, reaching up to 85.7% under the same experimental setting. However, faster R-CNN (73.5%), SSD (62.2%), YOLOv5x (84.6%), YOLOX (72.5%), YOLOv7 (81.3%), and the standard YOLOv8 (83.6%) display significant differences in terms of mAP, in comparison. The detection rate of the improved YOLOv8 is 106.4FPS, satisfying real-time detection requirements. Moreover, the proposed improved algorithm model in this study has parameters of 11.16M, which is considerably smaller than both faster R-CNN and YOLOv5x models and only slightly larger than the standard YOLOv8s model yet performing better in terms of detection precision and frame rate. In summary, the algorithm not only meets the requirements for real-time detection but also improves detection accuracy and possesses high versatility and practical value.

## 4. Discussion

### 4.1. Research Value

This research on the recognition of parental care behavior in *A. davidianus* using deep learning technology aims to overcome the limitations of traditional behavioral observation methods, capturing the details of *A. davidianus*’ behavior during the breeding period in an efficient and precise manner. It will objectively analyze and determine whether the parental care behaviors of *A. davidianus* are limited to behaviors, such as tail fanning, agitating, shaking, and egg eating [6,7], and explore the existence of other potential behaviors. In addition, the study will provide innovative technical means for observing the behavioral rhythms of *A. davidianus*, which will help to deeply explore the intrinsic connections between behavioral changes and environmental factors, such as revealing the specific behavioral patterns of *A. davidianus* when the dissolved oxygen content in the water decreases and the particular state of egg eating.

Through statistical analysis using behavioral recognition technology, we can quantify key indicators, such as the frequency and duration of *A. davidianus*’ parental care behaviors, thus fully grasping the characteristics of these behaviors. The acquisition of behavioral information will not only greatly promote the optimization of *A. davidianus*’ breeding techniques but also provide a scientific basis for targeted improvement and maintenance of the breeding environment for parental *A. davidianus*, thereby increasing the hatching success rate of the offspring and seeking welfare for the breeding of *A. davidianus*. This study has a positive guiding significance for the protection of *A. davidianus*, the maintenance of ecological balance, and the formulation of sustainable development strategies.

### 4.2. Limitation and Outlook

In this study, we collected relevant data by monitoring with infrared cameras installed above the cave entrances to construct a dataset of images depicting the brood care behavior of *A. davidianus*. However, the limited number of cameras and restricted monitoring angles often result in disproportionate representations of *A. davidianus* within the field of view. During the brooding period, *A. davidianus* tend to face outward with their heads and inward with their tails, persistently guarding the cave entrance and performing tail-fanning movements. The insufficient lighting at the bottom of the cave, which easily produces shadows, increases the difficulty of behavior recognition. For instance, the recognition accuracy of the tail fanning behavior in this study is relatively low. This situation is similar to that encountered in other studies of animal behavior recognition based on video [37,38].

Many scholars use 3D technology to overcome these limitations. Zhu et al. [39] proposed a video-based 3D monitoring system for aquatic animals. The system combines a catadioptric stereo-vision setup and robust tracking of a 3D motion-related behavior method to improve the recognition accuracy of swimming behavior of *Carassius auratus*, showing obvious advantages compared with the traditional 2D method. LiftPose3D is a technology that can convert the two-dimensional posture of laboratory animals into three-dimensional posture, which can achieve high-quality 3D pose estimation without complex camera arrays and tedious calibration procedures. The technique has been applied in many experimental systems such as flies, mice, rats, and macaques [40]. Wang et al. [41] has proposed an efficient 3D CNN algorithm that efficiently processes the spatial-temporal information of videos. This algorithm is capable of accurately and rapidly recognizing the basic motion behaviors of dairy cows in their natural environments, such as lying down, standing, walking, drinking, and feeding. In the future, we plan to introduce 3D recognition technology to optimize the model and improve the recognition accuracy of *A. davidianus*’ parental care behavior.

## 5. Conclusions

This study successfully developed an efficient automatic recognition algorithm for the parental care behavior of *A. davidianus* using deep learning-based object detection technology, addressing the time-consuming and labor-intensive limitations of traditional observation methods for such behavior. The experimental results show that the ML-YOLOv8 model has achieved 85.7% on the mAP50-95 metric, which is a 2.1% increase compared to the YOLOv8 model, while also reducing the computational and parameter requirements, and accurately identifying the parental care behaviors of *A. davidianus*. Additionally, in comparison with other mainstream object detection models such as Faster R-CNN, SSD, YOLOv5x, YOLOX, and YOLOv7, the ML-YOLOv8 model has respectively achieved performance improvements of 12.2%, 22.5%, 1.1%, 13.2%, and 4.4%. This innovative recognition method enhances both the accuracy and efficiency of identifying parental behaviors in *A. davidianus*. Additionally, it significantly contributes to optimizing breeding techniques and achieving intelligent breeding for *A. davidianus*.

## Figures and Tables

**Table 1:** Dataset classification of *A. davidianus*’ parental care behavior.

Behavior Types	Judging Standard	Label	Sample Size
Tail fanning	The tail of *A. davidianus* swings from side to side beside or in the egg pile.	shanwei	600
Agitating	The head of the *A. davidianus* drills into the egg pile or the body passes through the egg pile.	jiaodong	700
Shaking	The head or body of the *A. davidianus* straddles above or near the egg pile and swings from side to side or up and down.	zhendong	500
Egg eating	*A. davidianus* holds the egg with its mouth open, and it is often accompanied by shaking its head.	chsihi	700
Entering caves	Only the head of the *A. davidianus* appears near the cave mouth.	jindong	250
Exiting caves	Only the tail of the *A. davidianus* appears near the cave mouth.	chudong	250

**Table 2:** Training environment and hardware platform parameters table.

Category	Configuration
CPU	16 vCPU Intel(R) Xeon(R) Gold 6430
GPU	RTX A5000 24G
System environment	ubuntu20.04
Framework	Pytorch 1.11.0
Programming voice	Python 3.8

**Table 3:** Ablation experiment results.

Baseline	C2f-MSConv	SPPF-LSKA	WIoU	mAP@50-90	GFLOPs	Parameters/10^6^	FPS
YOLOv8s				83.6%	28.8	11.16	130.0
	√			84.9%	27.8	10.61	107.3
	√	√		85.4%	28.7	11.68	106.4
	√	√	√	85.7%	28.7	11.68	106.4

**Table 4:** Model comparison experiment results.

Model	mAP@50-95	GFLOPs	Parameters/10^6^	FPS
Faster-RCNN	73.5%	251.4	41.37	57.4
SSD	62.2%	62.7	24.26	48
YOLOv5x	84.6%	204.7	86.25	48.6
YOLOX	72.5%	26.8	9.00	83.3
YOLOv7	81.3%	103.5	37.62	56.4
YOLOv8s	83.6%	28.8	11.16	130.0
our	85.7%	28.7	11.68	106.4

## Data Availability

The data are not publicly available due to the privacy policy of the organization.

## Appendix A

**Table A1:** Component descriptions for YOLOv8 structure.

Component	Description
Conv	Convolutional layer that performs feature extraction using filters.
Conv2d	Two-dimensional convolutional layer crucial for image processing.
BatchNorm2d	Batch normalization for 2D layers, aiding in training stability and acceleration.
Bottleneck	Design pattern that temporarily reduces feature map dimensions to lower computational costs before restoring them.
C2f	Custom module in YOLOv8, likely involved in feature transformation or fusion.
Concat	Concatenation layer that merges feature maps to integrate multi-scale information.
SiLU	Sigmoid linear unit, an activation function that enhances deeper network training with self-gating.
Detect	The detection layer that outputs object localization and classification.
Upsample	Upsampling layer that increases feature map resolution for precise object localization.
SPPF	Module that captures multi-scale context through an efficient pyramid pooling technique
