# Correction: Li et al. Improved Chinese Giant Salamander Parental Care Behavior Detection Based on YOLOv8. *Animals* 2024, *14*, 2089

**DOI:** 10.3390/ani16081199

**Published:** 2026-04-15

**Authors:** Zhihao Li, Shouliang Luo, Jing Xiang, Yuanqiong Chen, Qinghua Luo

**Affiliations:** 1 School of Computer Science and Engineering, Jishou University, Zhangjiajie 427000, China; 2 Hunan Engineering Laboratory for Chinese Giant Salamander’s Resource Protection and Comprehensive Utilization, School of Biological Resources and Environmental Sciences, Jishou University, Zhangjiajie 427000, China; 3 Hunan Engineering Technology Research Center for Amphibian and Reptile Resource Protection and Product Processing, College of Biological and Chemical Engineering, Changsha University, Changsha 410022, China; 4 College of Biology and Pharmacy, Yulin Normal University, Yulin 537000, China; 5 School of Computer Science and Engineering, Central South University, Changsha 410017, China

## Error in Figure

In the original publication [1], there was a mistake in Figure 1. Simulate a natural *A. davidianus*’ breeding pond as published. This figure closely resembles a figure presented in our team’s earlier publication. (This remains the same and is not modified). The corrected Figure 1. Simulate a natural *A. davidianus*’ breeding pond appears below. 

In the original publication [1], there was a mistake in Figure 2. Example of *A. davidianus*’ parental care behavior dataset: (a) tail fanning; (b) agitating; (c) shaking; (d) egg eating; (e) entering caves; (f) exiting caves as published. The subfigure (b) labeled “agitating” bears similarity to that in a previously published paper by our team. Additionally, the label “male” in this subfigure should be revised to “head,” as it better reflects the context of the image. The corrected Figure 2. Example of *A. davidianus*’ parental care behavior dataset: (a) tail fanning; (b) agitating; (c) shaking; (d) egg eating; (e) entering caves; (f) exiting caves (This remains the same and is not modified.) appears below.

The authors state that the scientific conclusions are unaffected. This correction was approved by the Academic Editor. The original publication has also been updated.

## Figures and Tables

**Figure 1:**
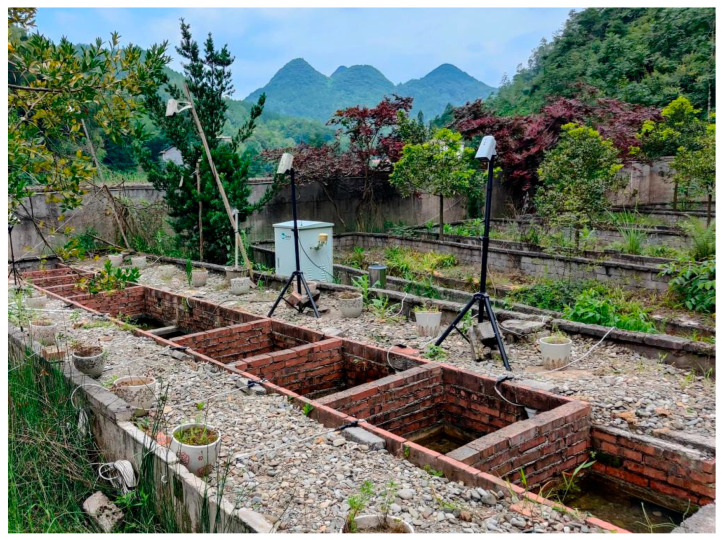
Simulate natural *A. davidianus*’ breeding pond.

**Figure 2:**
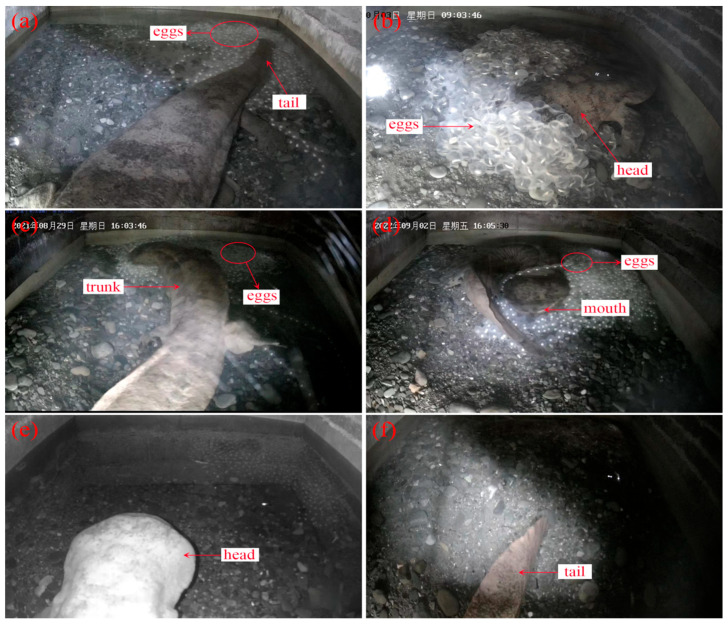
Example of *A. davidianus*’ parental care behavior dataset: (**a**) tail fanning; (**b**) agitating; (**c**) shaking; (**d**) egg eating; (**e**) entering caves; (**f**) exiting caves.
